# Supplementary material for: Human commensal gut Proteobacteria withstand type VI secretion attacks through immunity protein-independent mechanisms
Source: Nat Commun. 2021 Oct 1;12:5751. doi: 10.1038/s41467-021-26041-0 (PMC8486750; doi:10.1038/s41467-021-26041-0)
Supplement: Supplementary file 1 — Supplementary Information [file 41467_2021_26041_MOESM1_ESM.pdf]

SUPPLEMENTARY INFORMATION

Human commensal gut Proteobacteria withstand type VI secretion attacks through immunity protein-independent mechanisms

Nicolas Flaugnatti[1*], Sandrine Isaac[1*], Leonardo F. Lemos Rocha[1], Sandrine Stutzmann[1], Olaya Rendueles[2], Candice Stoudmann[1], Nina Vesel[1], Marc Garcia-Garcera[3], Amandine Buffet[2], Thibault G. Sana[1†], Eduardo P.C. Rocha[2] & Melanie Blokesch[1#]

[1] Laboratory of Molecular Microbiology, Global Health Institute, School of Life Sciences, Ecole Polytechnique Fédérale de Lausanne (EPFL), Lausanne, Switzerland

[2] Microbial Evolutionary Genomics, Institut Pasteur, CNRS, UMR3525, Paris, 75015, France.

[3] Department of Fundamental Microbiology, University of Lausanne, Lausanne, Switzerland

[*] These authors contributed equally: Nicolas Flaugnatti, Sandrine Isaac

[†] Present address: LIPME, Université de Toulouse, INRAE, CNRS, 31320 Castanet-Tolosan, France

[#] Correspondence to: melanie.blokesch@epfl.ch

Supplementary Figure S1

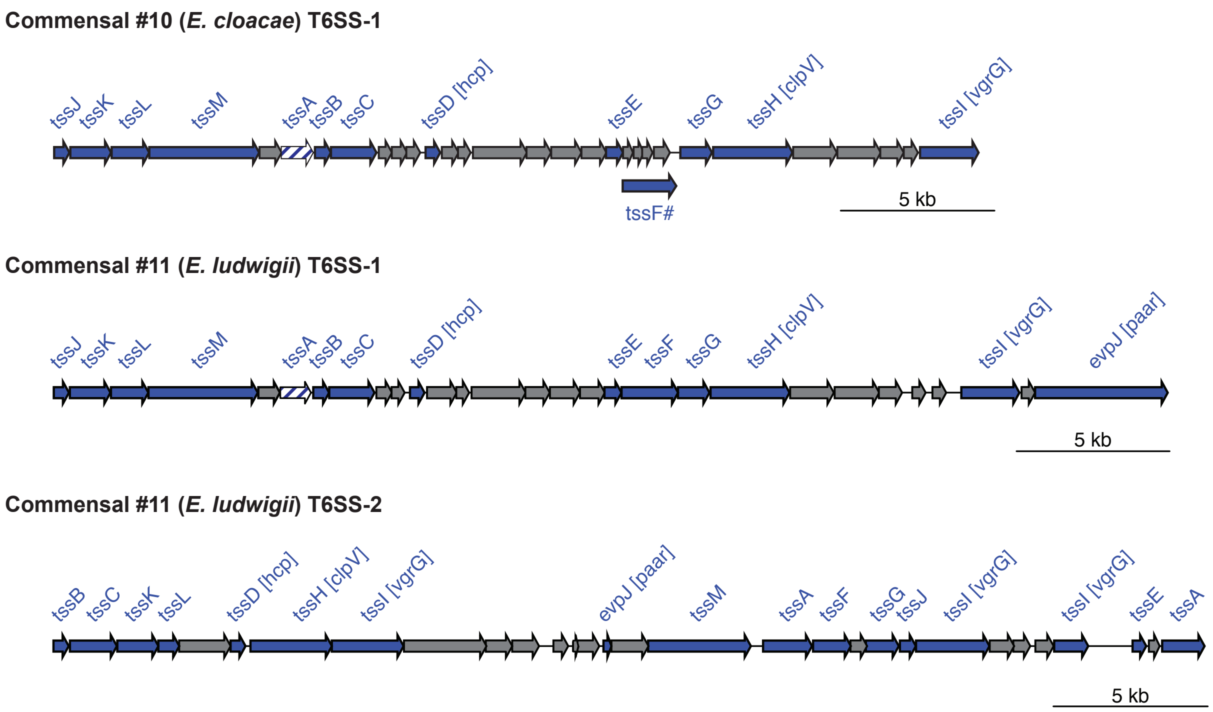

Fig. S1. Commensals belonging to the *Enterobacter cloacae* complex encode T6SS gene cluster(s). The genome sequences of the human commensal *Enterobacter* isolates #10 and #11 were inspected for the presence of T6SS gene cluster(s) using the program TXSScan. The core components of the T6SS detected with the program are indicated in blue. Manually annotated genes (*tssA*) are indicated by dashed arrows. #, indicates a region that was reassessed by Sanger sequencing, which revealed a sequencing artifact in the WGS data (details provided in Supplementary Data 5); the corrected non-interrupted *tssF* gene is depicted. Based on their gene organization, the clusters were classified as T6SS-1 or T6SS-2. Underlying source data are provided in the Source Data file and Supplementary Data files 4 and 5.

Supplementary Figure S2

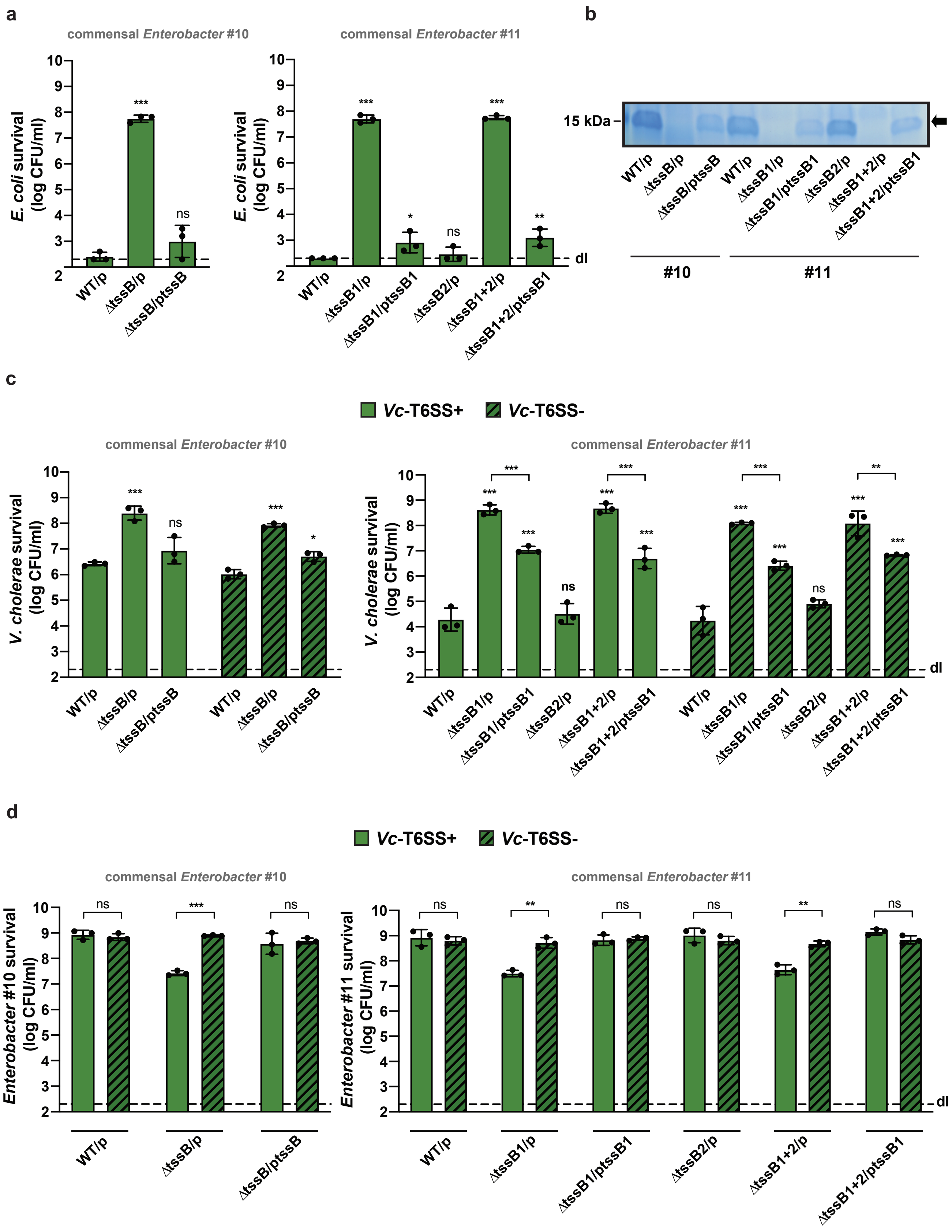

Fig. S2. ***Enterobacter*** **complex isolates kill** ***E. coli*** **and compete with** ***V. cholerae*** **in a T6SS-1-dependent manner.**

Survival of *E. coli* (a) or *V. cholerae* (c) was scored after coincubation with *Enterobacter* commensal strains #10 and #11 (carrying an empty plasmid [p], as control), their T6SS-1 and/or T6SS-2-negative mutants (ΔtssB for #10; ΔtssB1 and/or ΔtssB2 for #11; with empty plasmid [p]), or their T6SS-1-complemented derivates (containing plasmids ptssB or ptssB1). Prey survival values are indicated on the *Y*-axis. (b) Detection of secreted Hcp. Details as for Fig. 2. Expression of the plasmid-encoded genes was induced with 0.2% arabinose. Representative image (out of three independent experiments). (d) *Enterobacter* survival after co-incubation with T6SS-positive (plain bar) and T6SS-negative (dashed bar) *V. cholerae.* (a, c, and d) Values are derived from three independent experiments and the bars represent the mean (± SD, as shown by the error bars). dl, detection limit, as indicated by the dashed line. Significant differences were determined using a one-way ANOVA followed by Holm-Sidak's multiple comparison test. Statistical significance indicated above each bar reflects comparison to the corresponding WT sample of each group (WT/p), except where comparison groups are explicitly shown by the brackets (a, c). For panel d, significant differences between samples containing T6SS+ and T6SS- predators were determined using a two-sided Student's *t*-test corrected for multiple comparisons. *, $p < 0.05$; **, $p < 0.01$; ***, $p < 0.001$; ns, not significant. Source data underlying all panels are provided in the Source Data file.

Supplementary Figure S3

Commensal #8 (*Klu. cryocrescens*) T6SS-3

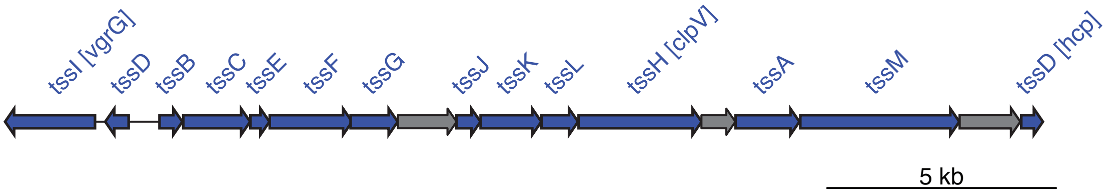

Commensal #9 (*Klu. cryocrescens*) T6SS-3

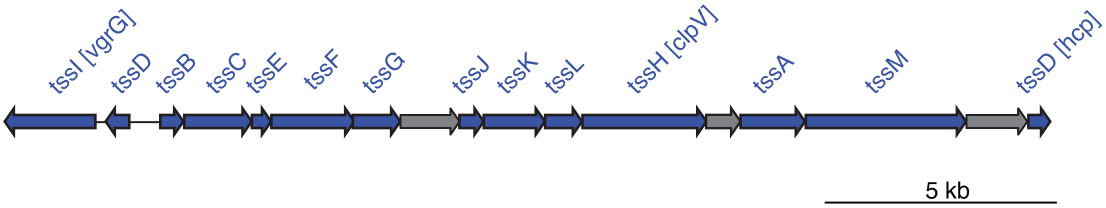

Fig. S3. Commensal *Kluyvera cryocrescens* strains harbor a T6SS-3 gene cluster. A third T6SS gene cluster (T6SS-3) with a different gene order compared to the T6SS-1- and T6SS-2 clusters was identified in the *Kluyvera cryocrescens* commensals #8 and #9. Underlying source data are provided in the Source Data file and Supplementary Data files 2 and 5.

Supplementary Figure S4

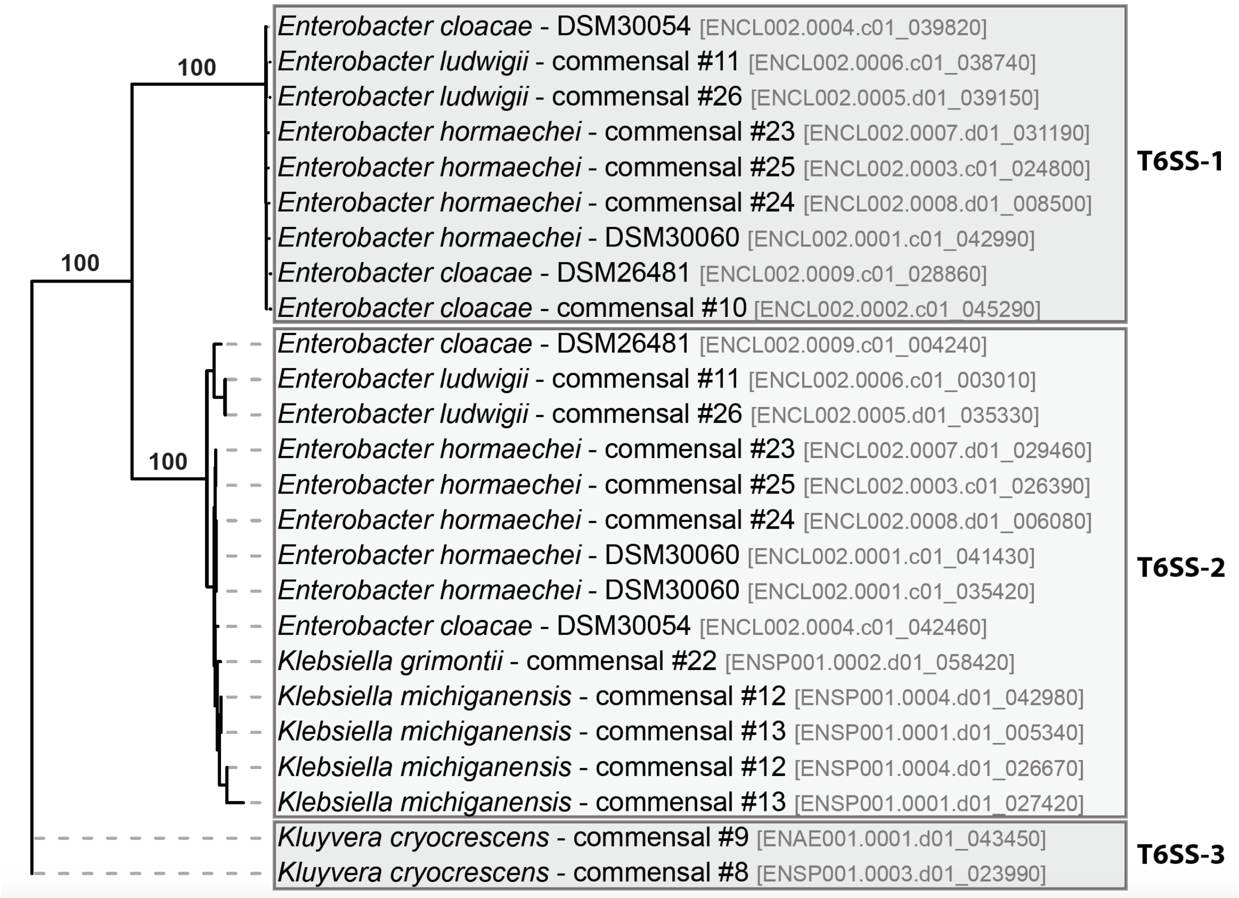

Fig. S4. TssB/TssC phylogenetic tree supports the presence of three distinctives T6SS clusters. A phylogenetic tree was constructed based on the concatenated alignments of TssB and TssC protein sequences from the T6SS clusters. Bootstrap values are indicated for the main branches. The ID names (in light gray) correspond to the individual TssB proteins (see Supplementary Data 5 and 7 for details). Underlying source data are provided in the Source Data file and Supplementary Data files 2, 4, and 5.

Supplementary Figure S5

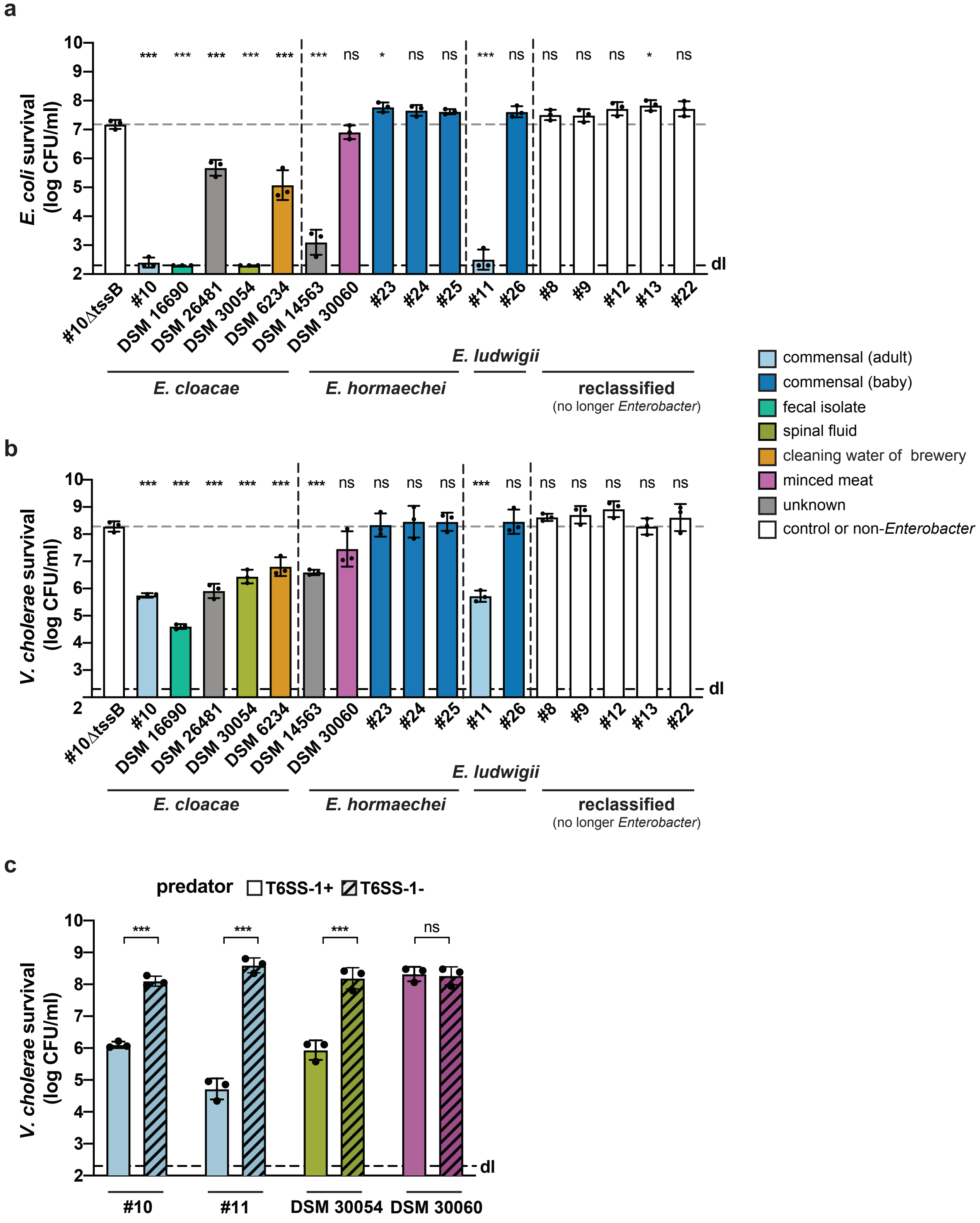

Fig. S5. A subset of *E. cloacae* complex strains kills *E. coli* and *V. cholerae* in a T6SS-1-dependent manner. Survival of *E. coli* (a) or *V. cholerae* (b, c) was scored after coincubation with a collection of *E. cloacae* complex strains (entire set compared to the selection shown in Fig. 2), including initially misclassified isolates (#8, #9, #12, #13, #22), and selected T6SS-1-negative derivatives (panel c). Values are derived from three independent experiments and the bars represent the mean (± SD, as shown by the error bars). dl, detection limit, as indicated by the black dashed line. Significant differences were determined using a one-way ANOVA followed by Holm-Sidak's multiple comparison test comparing each strain to the T6SS-deficient control commensal strain (#10ΔtssB; value indicated by the dotted gray line) (a, b) or using a two-sided Student's *t*-test corrected for multiple comparison (c). *, $p < 0.05$, **, $p < 0.01$; ***, $p < 0.001$; ns, not significant. The colors reflect the origin of the isolates, as shown by the legend on the right of the figure. Source data underlying all panels are provided in the Source Data file.

Supplementary Figure S6

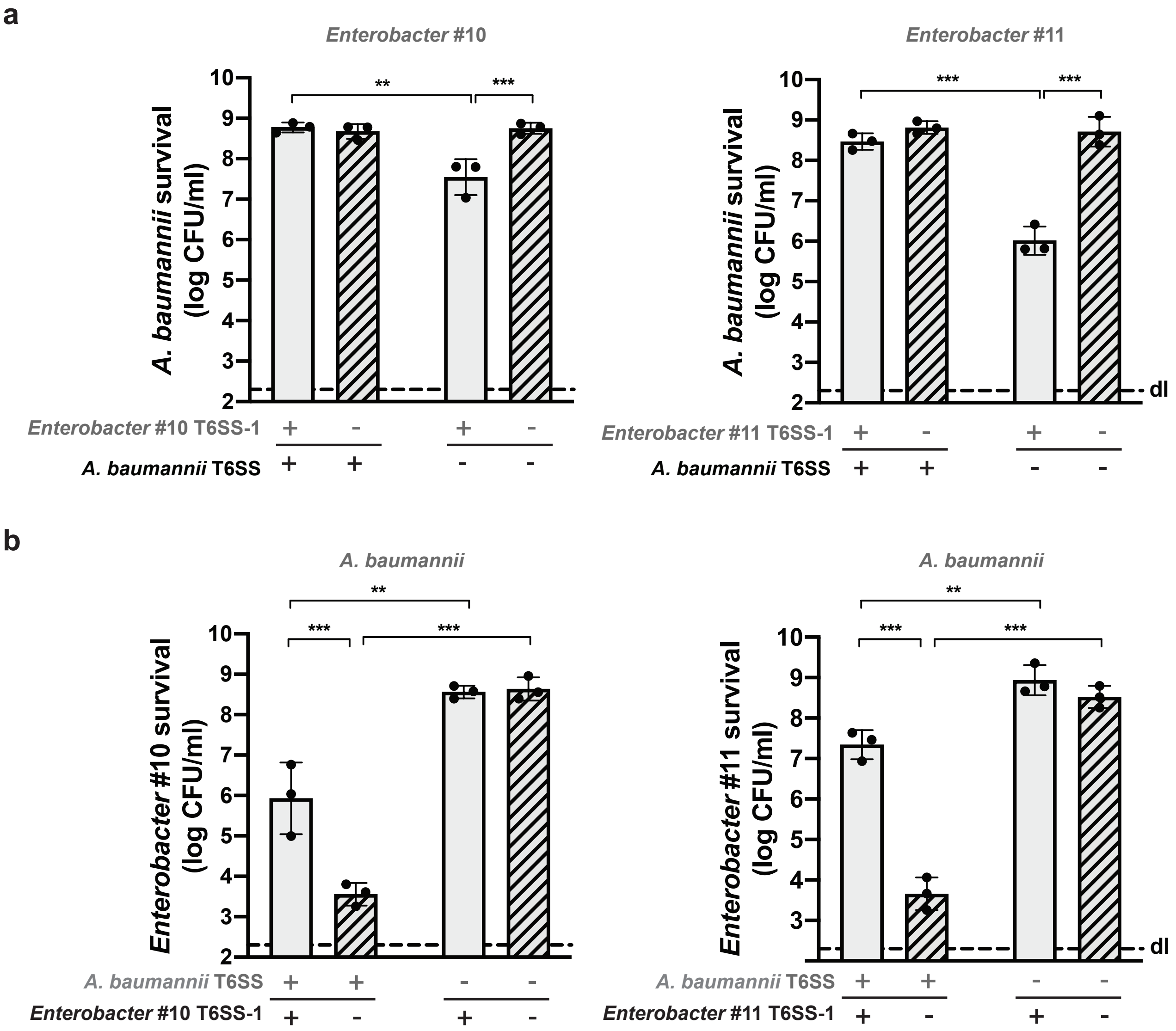

Fig. S6. *Acinetobacter baumannii* and commensal *Enterobacter* isolates compete in a T6SS-dependent manner. Wild-type (T6SS+) and T6SS-deficient (T6SS-) *A. baumannii* were co-incubated with WT *Enterobacter* isolates #10 and #11 or their T6SS-1-deficient mutants (#10ΔtssB and #11ΔtssB1). The survival of *A. baumannii* (a) and *Enterobacter* (b) was scored as indicated on the *Y*-axis. Values are derived from three independent experiments and the bars represent the mean (± SD, as defined by the error bars). Significant differences were determined using a two-sided Student's *t*-test corrected for multiple comparisons. Only statistically significant differences are shown. **, $p < 0.01$; ***, $p < 0.001$. Source data underlying all panels are provided in the Source Data file.

Supplementary Figure S7

a

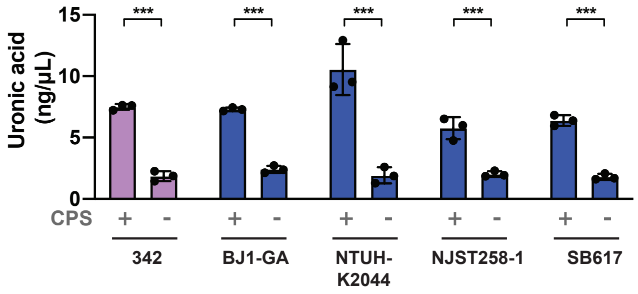

Strain	K locus	Biochemical structure
342<br>BJ1-GA<br>NTUH-K2044	KL30<br>KL2<br>KL1	Defined
NJST258-1<br>SB617	KL107<br>KL124[(§)]	Unknown

b

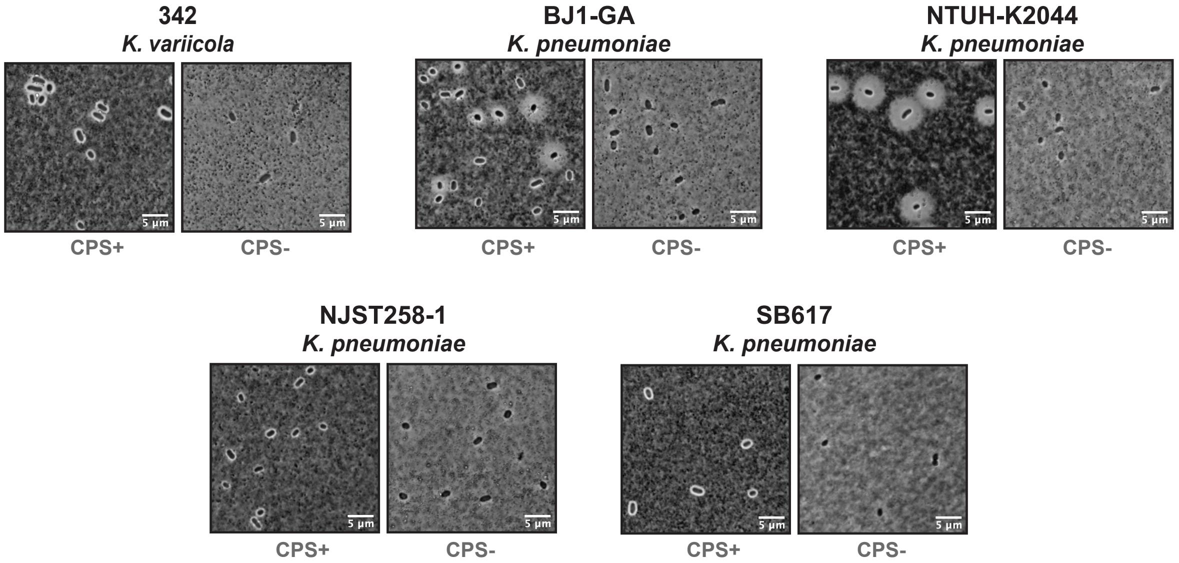

c

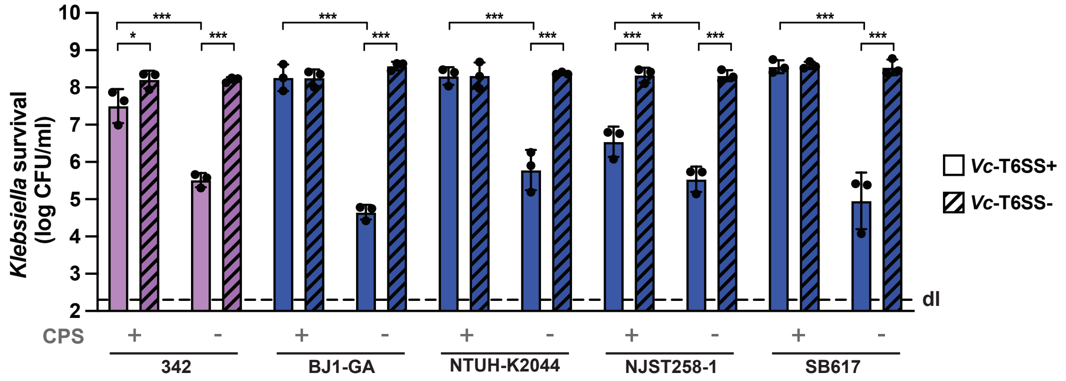

d

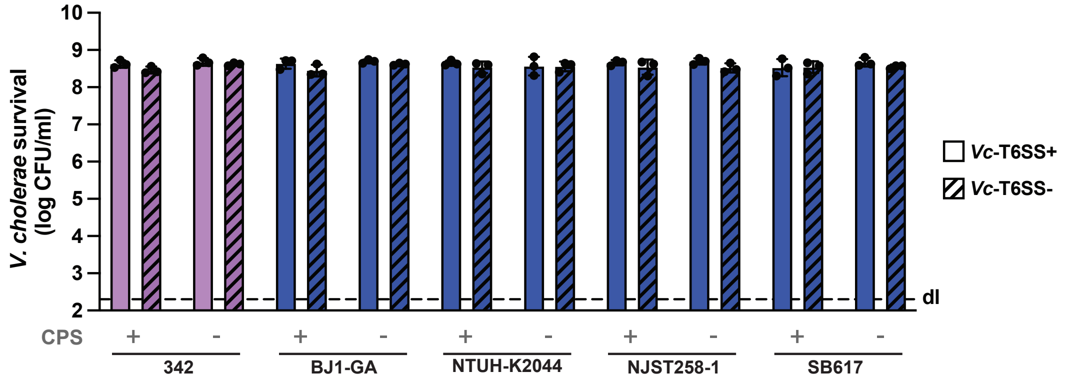

Fig. S7. Group I capsule protect *Klebsiella* species against *V. cholerae*'s T6SS attacks. (a) Deletion of the capsule biosynthesis gene *wza* eliminates capsule formation in well-studied *Klebsiella* strains with different K serotypes. Group I capsules were scored by uronic acid quantification in the CPS+ (*wza*-positive) and CPS- (*wza*-negative) bacteria. The table indicates the K antigen prediction by the Kaptive program. (§), low confidence prediction. (b) CPS+ and CPS- strains (Δwza) were imaged after India ink staining. Representative images are shown. Scale bar, 5µm. (c) Non-encapsulated strains are susceptible to *V. cholerae*'s T6SS assaults. The CPS+ and their polysaccharide export impaired CPS- *Klebsiella* strains were tested for survival against T6SS+ (WT; plain bars) or T6SS- (ΔvipA; stripped bars) *V. cholerae*, as indicated on the *Y*-axis. (d) *V. cholerae* is not killed by *Klebsiella* strains. T6SS+ (WT; plain bars) or T6SS- (ΔvipA; stripped bars) *V. cholerae* were tested for survival against CPS+ and their polysaccharide export impaired CPS- *Klebsiella* strains as indicated on the *X*-axis. Panels a, c and d: Values are derived from three independent experiments and the bars represent the mean (± SD, as defined by the error bars). Significant differences were determined using a two-sided Student's *t*-test corrected for multiple comparisons. Only significant differences within each *Klebsiella* strain group (as shown below the X-axis) are indicated. *, $p < 0.05$; **, $p < 0.01$; ***, $p < 0.001$. dl, detection limit, as indicated by the dashed line. Source data underlying all panels are provided in the Source Data file.

Supplementary Figure S8

a

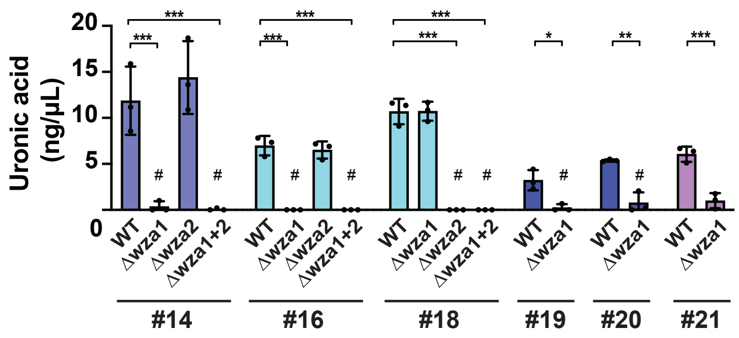

b

Commensal	Species	Predicted K serotype	*wza* in capsule cluster
#14	*K. michiganensis*	-	*wza1*
#16	*K. oxytoca*	-	*wza1*
#18	*K. oxytoca*	-	*wza2*
#19	*K. pneumoniae*	KL31	*wza1*
#20	*K. pneumoniae*	KL31	*wza1*
#21	*K. variicola*	KL61	*wza1*

c

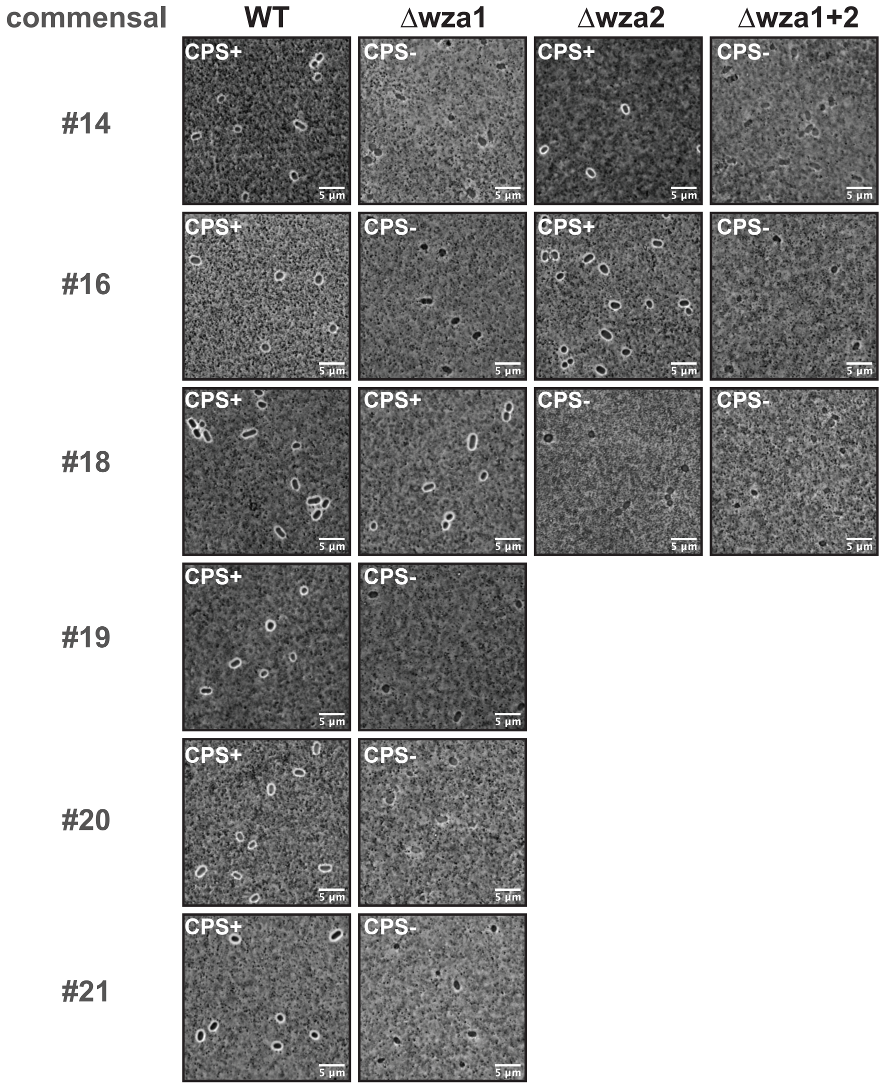

Fig. S8. Deletion of *wza* in the capsular biosynthesis gene cluster blocks capsule production in commensal *Klebsiella* strains. (a) The production of group I capsules by the *Klebsiella* gut commensal isolates was assessed by quantification of the strains' uronic acid content. The color code as well as the *X*-axis labels are as in Fig. 1. (b) Predicted K antigens of commensal *Klebsiella* isolates. The table indicates the *in silico* predicted K antigen (-, unpredictable) and which *wza* gene is located in the capsular operon. (c) Capsule visualization of *Klebsiella* WT (CPS+) and *wza*-minus strains after India Ink staining. Representative images are shown. Scale bar, 5μm. For panel a, values are derived from three independent experiments and the bars represent the mean (± SD, as defined by the error bars). #, at least one sample was below the detection limit. Significant differences were determined using a two-sided Student's *t*-test corrected for multiple comparisons. Only significant differences compared to the corresponding WT strain are indicated. *, $p < 0.05$; **, $p < 0.01$; ***, $p < 0.001$. Source data underlying all panels are provided in the Source Data file.

Supplementary Figure S9

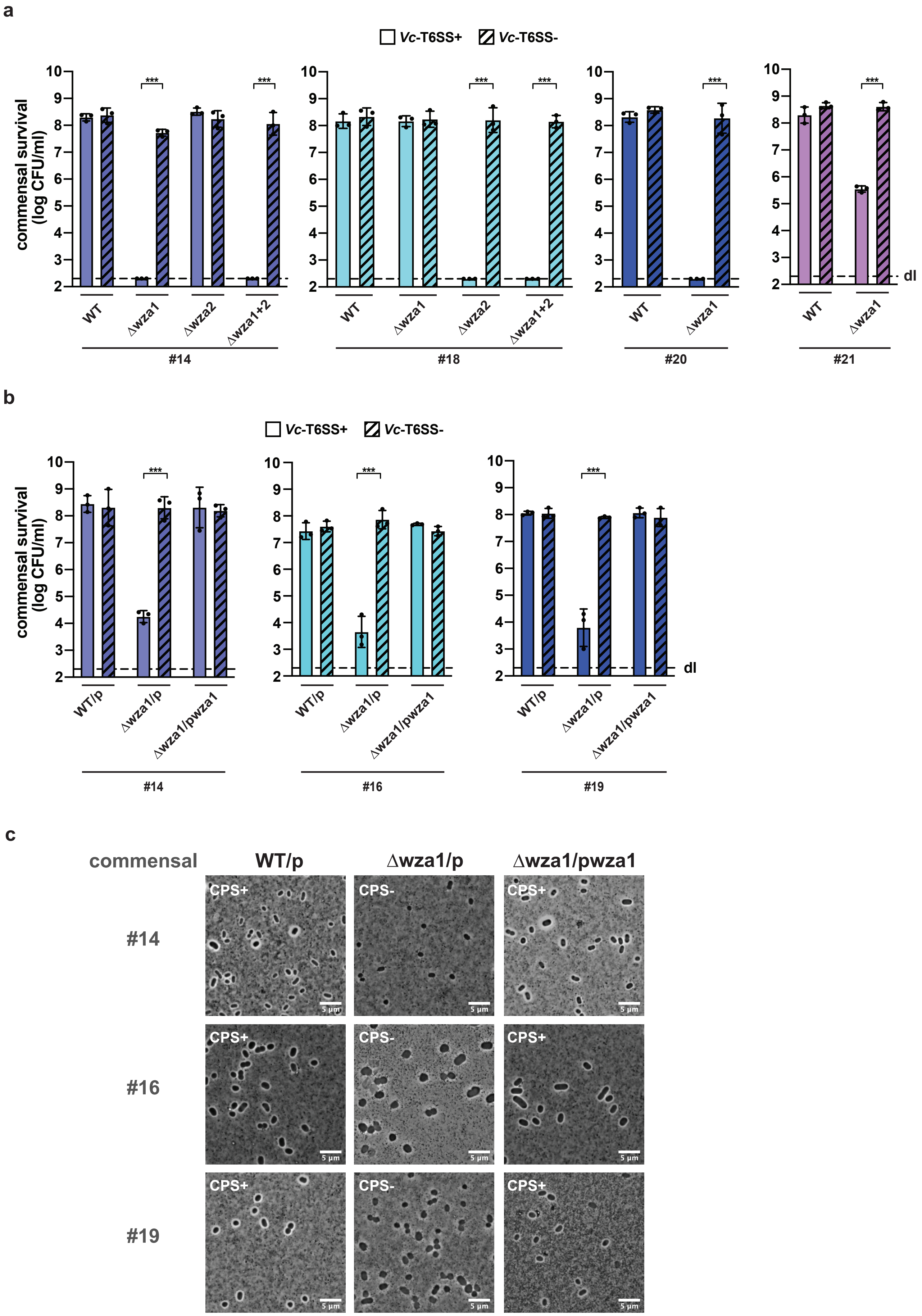

Fig. S9. Group I capsule protects against T6SS attacks from *V. cholerae*. (a) Extended set of representative WT and polysaccharide export protein-negative mutants (Δwza1 and/or Δwza2) of *Klebsiella* commensal strains (#14, #18, #20, #21) showing their survival after coincubation with T6SS+ (plain bars) or T6SS- (ΔvipA; stripped bars) *V. cholerae* (as for #16 and #19 in Fig. 4c). (b and c) Complementation of capsule-deficient mutant strains. The capsule-deficient mutants of the commensal *Klebsiella* strains #14, #16, #19 were complemented with their respective *wza1* gene copy on a plasmid *in trans* (pwza1). The complemented strains as well as the vector controls of the WT and mutant strains (containing the empty plasmid [p]) were tested for their survival in the presence of T6SS+ (WT; plain bars) or T6SS- (ΔvipA; stripped bars) *V. cholerae* (panel b) or imaged after Indian Ink staining to check their capsulation status (panel c). Scale bar, 5µm. dl, detection limit, as indicated by the dashed line. Panels a-b: Values are derived from three independent experiments and the bars represent the mean (± SD, as defined by the error bars). Significant differences were determined using a two-sided Student's *t*-test corrected for multiple comparisons. Only significant differences are indicated. ***, $p < 0.001$. Source data underlying all panels are provided in the Source Data file.

Supplementary Figure S10

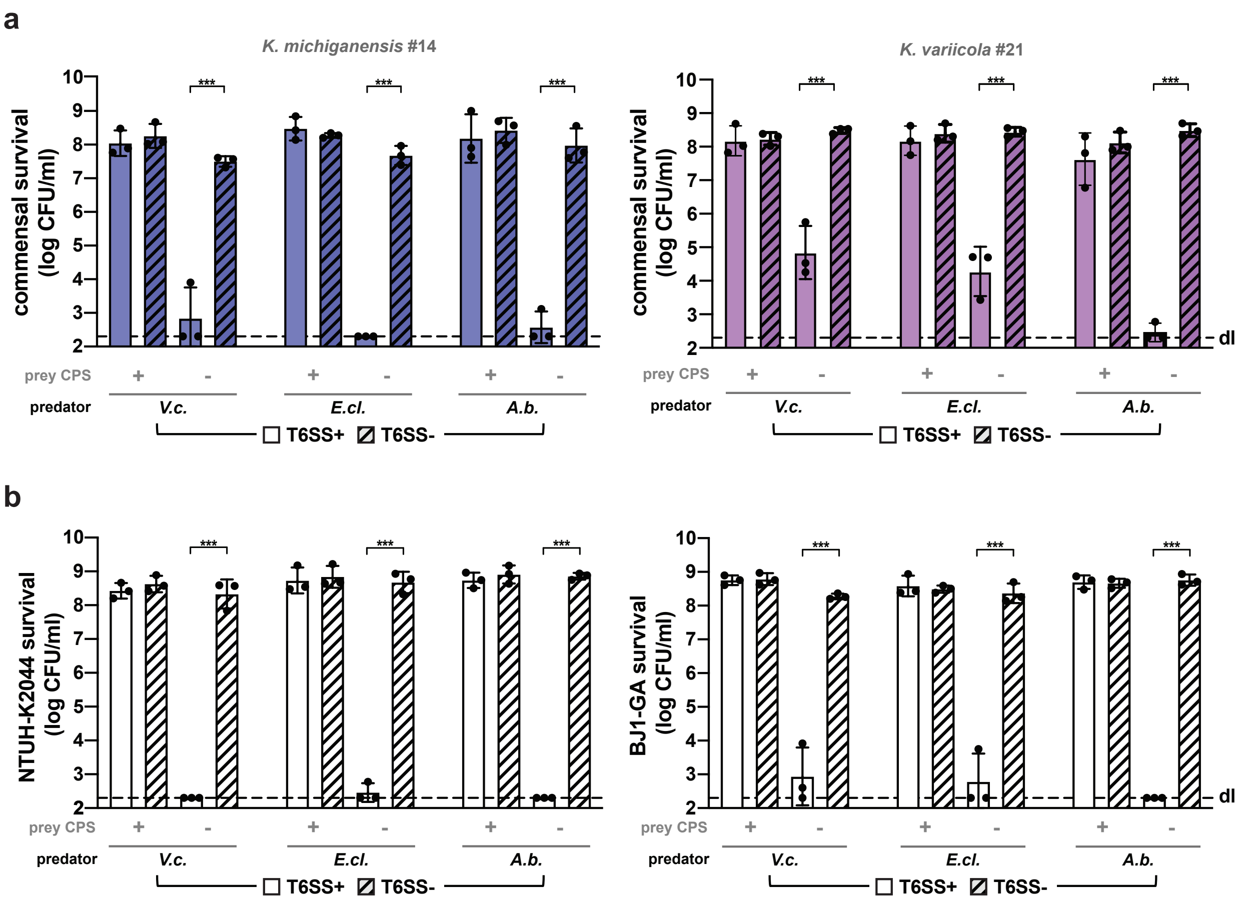

Fig. S10. Group I capsule protects *Klebsiella* species against T6SS attacks from pathogenic bacteria.

Additional examples of commensal *Klebsiella* WT (*K. michiganensis* #14 and *K. variicola* #21) and *wza*-negative mutants (a) or the well-studied CPS-positive (CPS+) *K. pneumoniae* strains NTUH-K2044 and BJ1-GA and their CPS-negative (CPS-) mutants (b) were tested for their survival in the presence of diverse T6SS+ (WT; plain bars) or T6SS- (stripped bars) pathogenic bacteria (*V. cholerae* [*V.c.*], *E. cloacae* [*E.cl.*; commensal #10], or *A. baumannii* [*A.b.;* strain A118]). Values are derived from three independent experiments and the bars represent the mean (± SD, as defined by the error bars). dl, detection limit, as indicated by the dashed line. Only significant differences are indicated. Significance was determined using a Student's *t*-test corrected for multiple comparisons. ***, $p < 0.001$. Source data underlying all panels are provided in the Source Data file.

Supplementary References (Covering Supplementary Information and Data files)

1. Aldova E, Laznickova K, Stepankova E, Lietava J. Isolation of nonagglutinable vibrios from an enteritis outbreak in Czechoslovakia. *J Infect Dis* **118**, 25-31 (1968).
2. Blokesch M, Schoolnik GK. Serogroup conversion of *Vibrio cholerae* in aquatic reservoirs. *PLoS Pathog* **3**, e81 (2007).
3. Van der Henst C*, et al.* Molecular insights into *Vibrio cholerae*'s intra-amoebal host-pathogen interactions. *Nat Commun* **9**, 3460 (2018).
4. de Sousa JAM, Buffet A, Haudiquet M, Rocha EPC, Rendueles O. Modular prophage interactions driven by capsule serotype select for capsule loss under phage predation. *ISME J* **14**, 2980–2996 (2020).
5. Buffet A, Rocha EPC, Rendueles O. Nutrient conditions are primary drivers of bacterial capsule maintenance in *Klebsiella*. *Proc Biol Sci* **288**, 20202876 (2021).
6. Escobar-Paramo P*, et al.* Identification of forces shaping the commensal *Escherichia coli* genetic structure by comparing animal and human isolates. *Environ Microbiol* **8**, 1975-1984 (2006).
7. Rendueles O*, et al.* Screening of *Escherichia coli* species biodiversity reveals new biofilm-associated antiadhesion polysaccharides. *mBio* **2**, e00043-11 (2011).
8. Simon R, Priefer U, Pühler A. A broad host range mobilization system for *in vivo* genetic engineering: transposon mutagenesis in Gram negative bacteria. *Nat Biotechnol* **1**, 784-791 (1983).
9. Ferrières L*, et al.* Silent mischief: bacteriophage Mu insertions contaminate products of *Escherichia coli* random mutagenesis performed using suicidal transposon delivery plasmids mobilized by broad-host-range RP4 conjugative machinery. *J Bacteriol* **192**, 6418-6427 (2010).
10. Metzger LC, Stutzmann S, Scrignari T, Van der Henst C, Matthey N, Blokesch M. Independent Regulation of Type VI Secretion in *Vibrio cholerae* by TfoX and TfoY. *Cell Rep* **15**, 951-958 (2016).
11. Vial PA*, et al.* Characterization of enteroadherent-aggregative *Escherichia coli,* a putative agent of diarrheal disease. *J Infect Dis* **158**, 70-79 (1988).
12. Swords WE, Cannon BM, Benjamin WH, Jr. Avirulence of LT2 strains of *Salmonella typhimurium* results from a defective *rpoS* gene. *Infect Immun* **65**, 2451-2453 (1997).
13. Vesel N, Blokesch M. Pilus Production in *Acinetobacter baumannii* Is Growth Phase Dependent and Essential for Natural Transformation. *J Bacteriol* **203**, e00034-00021 (2021).
14. Forster SC*, et al.* A human gut bacterial genome and culture collection for improved metagenomic analyses. *Nat Biotechnol* **37**, 186-192 (2019).
15. Shao Y*, et al.* Stunted microbiota and opportunistic pathogen colonization in caesarean-section birth. *Nature* **574**, 117-121 (2019).
16. Meibom KL, Li XB, Nielsen AT, Wu CY, Roseman S, Schoolnik GK. The *Vibrio cholerae* chitin utilization program. *Proc Natl Acad Sci USA* **101**, 2524-2529 (2004).
17. Metzger LC, Matthey N, Stoudmann C, Collas EJ, Blokesch M. Ecological implications of gene regulation by TfoX and TfoY among diverse *Vibrio* species. *Environ Microbiol* **21**, 2231-2247 (2019).
18. Guzman LM, Belin D, Carson MJ, Beckwith J. Tight regulation, modulation, and high-level expression by vectors containing the arabinose P_{BAD} promoter. *J Bacteriol* **177**, 4121-4130 (1995).
19. Seitz P*, et al.* ComEA Is Essential for the Transfer of External DNA into the Periplasm in Naturally Transformable *Vibrio cholerae* Cells. *PLoS Genet* **10**, e1004066 (2014).

20. Wu KM, *et al.* Genome sequencing and comparative analysis of *Klebsiella pneumoniae* NTUH-K2044, a strain causing liver abscess and meningitis. *J Bacteriol* **191**, 4492-4501 (2009).
21. Bialek-Davenet S, Leflon-Guibout V, Tran Minh O, Marcon E, Moreau R, Nicolas-Chanoine MH. Complete deletion of the ramR gene in an *in vitro*-selected mutant of *Klebsiella pneumoniae* overexpressing the AcrAB efflux pump. *Antimicrob Agents Chemother* **57**, 672-673 (2013).
22. Fouts DE*, et al.* Complete genome sequence of the N2-fixing broad host range endophyte *Klebsiella pneumoniae* 342 and virulence predictions verified in mice. *PLoS Genet* **4**, e1000141 (2008).
23. Deleo FR, et al. Molecular dissection of the evolution of carbapenem-resistant multilocus sequence type 258 *Klebsiella pneumoniae*. *Proc Natl Acad Sci* USA **111**, 4988-4993 (2014).
24. Blin C, Passet V, Touchon M, Rocha EPC, Brisse S. Metabolic diversity of the emerging pathogenic lineages of *Klebsiella pneumoniae*. *Environ Microbiol* **19**, 1881-1898 (2017).
25. Ren Y, et al. Complete genome sequence of *Enterobacter cloacae* subsp. cloacae type strain ATCC 13047. *J Bacteriol* **192**, 2463-2464 (2010).
