# Supplementary material for: Human commensal gut Proteobacteria withstand type VI secretion attacks through immunity protein-independent mechanisms
Source: Nat Commun. 2021 Oct 1;12:5751. doi: 10.1038/s41467-021-26041-0 (PMC8486750; doi:10.1038/s41467-021-26041-0)
Supplement: Supplementary file 3 — Supplementary data file legends [file 41467_2021_26041_MOESM3_ESM.docx]

**Description of Additional Supplementary Files**

**File Name: Supplementary Data 1**

Description: Bacterial strains and plasmids used in this study.

**File Name: Supplementary Data 2**

Description: List with detailed information on commensal isolates and DSMZ strains including genome accession numbers and initial and revised species classifications.

**File Name: Supplementary Data 3**

Description: List of core-genome gene families.

**File Name: Supplementary Data 4**

Description: Details on whole-genome sequencing data and NCBI accession numbers.

**File Name: Supplementary Data 5**

Description: Details on T6SS clusters detected using TXSScan.

**File Name: Supplementary Data 6**

Description: Nucleotide sequence of the T6SS genes indicated in Supplementary Data 5.

**File Name: Supplementary Data 7**

Description: Protein sequences of translated T6SS genes indicated in Supplementary Data 5.

**File Name: Supplementary Data 8**

Description: Analysis of the survival to T6SS assaults as a function of the capsule group.

**File Name: Supplementary Data 9**

Description: List of *Klebsiella* clinical and environmental isolate strains and *Enterobacter cloacae* type strain ATCC 13047 and their genome accession numbers.

**File Name: Supplementary Data 10**

Description: List of primers used in this study.

**File Name: Supplementary Data 11**

Description: List of statistical analyses with *P* values.
