# Supplementary material for: Human commensal gut Proteobacteria withstand type VI secretion attacks through immunity protein-independent mechanisms
Source: Nat Commun. 2021 Oct 1;12:5751. doi: 10.1038/s41467-021-26041-0 (PMC8486750; doi:10.1038/s41467-021-26041-0)
Supplement: Supplementary file 15 — Reporting Summary [file 41467_2021_26041_MOESM15_ESM.pdf]

Corresponding author(s): Melanie Blokesch

Last updated by author(s): Sep 3, 2021

Reporting Summary

Nature Research wishes to improve the reproducibility of the work that we publish. This form provides structure for consistency and transparency in reporting. For further information on Nature Research policies, see our Editorial Policies and the Editorial Policy Checklist.

Statistics

For all statistical analyses, confirm that the following items are present in the figure legend, table legend, main text, or Methods section.

n/a	Confirmed	
☐	☒	The exact sample size (n) for each experimental group/condition, given as a discrete number and unit of measurement
☐	☒	A statement on whether measurements were taken from distinct samples or whether the same sample was measured repeatedly
☐	☒	The statistical test(s) used AND whether they are one- or two-sided<br>*Only common tests should be described solely by name; describe more complex techniques in the Methods section.*
☒	☐	A description of all covariates tested
☐	☒	A description of any assumptions or corrections, such as tests of normality and adjustment for multiple comparisons
☐	☒	A full description of the statistical parameters including central tendency (e.g. means) or other basic estimates (e.g. regression coefficient) AND variation (e.g. standard deviation) or associated estimates of uncertainty (e.g. confidence intervals)
☐	☒	For null hypothesis testing, the test statistic (e.g. F, t, r) with confidence intervals, effect sizes, degrees of freedom and P value noted<br>*Give P values as exact values whenever suitable.*
☒	☐	For Bayesian analysis, information on the choice of priors and Markov chain Monte Carlo settings
☒	☐	For hierarchical and complex designs, identification of the appropriate level for tests and full reporting of outcomes
☒	☐	Estimates of effect sizes (e.g. Cohen's d, Pearson's r), indicating how they were calculated

Our web collection on statistics for biologists contains articles on many of the points above.

Software and code

Policy information about availability of computer code

Data collection	Microscopy data were collected using the ZEN BLUE 2.6 software from Zeiss. For genome sequences, the pooled bar-coded DNA libraries were sequenced with v3.0/v3.0 chemistry and diffusion loading on a PacBio Sequel instrument (Pacific Biosciences, Menlo Park, CA, USA) at 600 min movie length, pre-extension time of 120 min, using one SMRT cell 1M v3.
Data analysis	Genome assemblies were done using CANU v. 2.0 and the circularization of the genomes was improved using Circlator v.1.5.5. Genomes were annotated by the NCBI Prokaryotic Genome Annotation Pipeline (version 4.11).<br><br>Kleborate v. 0.4.0b was used for taxonomic reclassification (mainly for Klebsiella species).<br>The software OPSCAN v.0.1. was used to determine the core-genomes following a previously published approach (https://bioinfo.mnhn.fr/abi/public/opscan/).<br><br>Multi sequence alignment and trimming were performed using the program MAFFT version 7.453 and trimAl v1.4.rev15. Phylogenetic trees were inferred using IQ-TREE v.2.0.4 and visualized with iTol v5.5.1.<br><br>Prokka version v.1.14.6 was used, where indicated in the manuscript.<br><br>The presence of putative T6SS operons was determined using the the module TXSScan v.1 from MacSyFinder v.2. The identified clusters were represented using the R package genoPlotR v. 0.8.9.<br><br>The presence of putative capsule biogenesis operons was determined using CapsuleFinder v.1. The presence of the different capsule types in each isolate was represented using the R packages ggplot2 v.3.0.0, RColorBrewer v.1.1-2, and the function heatmap.2 from the package gplots v.3.0.1.1.

Statistical analyses were performed using GraphPad Prism 8.4.2 and 9.0.2 (GraphPad Software, Inc., CA, US) and JMP® 13.2.0 (SAS Institute Inc.).

Microscopy images were analyzed using ImageJ v2.0.0-rc-69/1.52p.

For manuscripts utilizing custom algorithms or software that are central to the research but not yet described in published literature, software must be made available to editors and reviewers. We strongly encourage code deposition in a community repository (e.g. GitHub). See the Nature Research guidelines for submitting code & software for further information.

Data

Policy information about availability of data

All manuscripts must include a data availability statement. This statement should provide the following information, where applicable:

- Accession codes, unique identifiers, or web links for publicly available datasets
- A list of figures that have associated raw data
- A description of any restrictions on data availability

All data generated or analyzed during this study are included in this published article (and its Supplementary Information and Supplementary Data files). Source data are provided with this paper. Accession number for whole-genome sequencing data are provided in the text and in Supplementary Data files 2 and 4. The PacBio raw read data of the five whole-genome sequenced Enterobacter strains generated on this study have been deposited in the NCBI's Sequence Read Archive (SRA) database under the Bioproject accession number PRJNA640151 [https://www.ncbi.nlm.nih.gov/bioproject/?term=PRJNA640151]. Details on the SRA accession numbers, BioSample accession numbers, and individual genome accession numbers of the de-novo-assembled and circularized genomes are provided in Supplementary Data file 4.

Field-specific reporting

Please select the one below that is the best fit for your research. If you are not sure, read the appropriate sections before making your selection.

☒ Life sciences ☐ Behavioural & social sciences ☐ Ecological, evolutionary & environmental sciences

For a reference copy of the document with all sections, see nature.com/documents/nr-reporting-summary-flat.pdf

Life sciences study design

All studies must disclose on these points even when the disclosure is negative.

Sample size	Sample sizes were according to those strains included in each experiment and not predetermined. This is state-of-the-art for genetic studies. 'n' values indicate biologically independent replicates (see below).
Data exclusions	No data were excluded from the analyses if not explicitly stated (e.g., correlation analysis excluded E. cloacae strains, as mentioned in the text, given that the reason for it's T6SS protection was already established in the first part of the manuscript).
Replication	Experiments were performed at least three independent times (biologically independent experiments), as indicated in the legends.
Randomization	Not performed, as not applicable for this in vitro study.
Blinding	Not performed, as not applicable for this in vitro study.

Reporting for specific materials, systems and methods

We require information from authors about some types of materials, experimental systems and methods used in many studies. Here, indicate whether each material, system or method listed is relevant to your study. If you are not sure if a list item applies to your research, read the appropriate section before selecting a response.

Materials & experimental systems

n/a	Involved in the study	
☒	☐	Antibodies
☒	☐	Eukaryotic cell lines
☒	☐	Palaeontology and archaeology
☒	☐	Animals and other organisms
☒	☐	Human research participants
☒	☐	Clinical data
☒	☐	Dual use research of concern

Methods

n/a	Involved in the study	
☒	☐	ChIP-seq
☒	☐	Flow cytometry
☒	☐	MRI-based neuroimaging
